# Trans-active factors controlling the IL-2 gene in adult human T-cell subsets

**DOI:** 10.1155/S0962935192000073

**Published:** 1992

**Authors:** A. Mouzaki, R. H. Zubler, A. Doucet, D. Rungger

**Affiliations:** 1 Haematology Geneva University Hospital 25, rue Micheli-du-Crest Geneva 4 CH-1211 Switzerland; 2 Station de Zoologie expérimentale University of Geneva 154, rte de Malagnou Chêne- Bougeries CH-1224 Switzerland

## Abstract

IL-2 secretion in total or subsets of PHA/PMA-stimulated PBMC-derived human T-lymphocytes was monitored and found to be largely due to CD4^+^CD8^−^ cells. The presence and functional state of transcription factors (TF) was assessed by protein-DNA interaction assays and functional transactivation experiments in the Xenopts oocyte system, modulating IL-2 transcription by injection of proteins. The results reveal that CD4^+^CD8^−^ cells contain both, functional silencer in their resting, and positive TF in their activated states while the CD4^+^CD8^−^ group contains only non-functional positive TF. This demonstrates that the on/off switch of IL-2 transcription is based on the same mechanism in primary T-lymphocytes of mouse spleen and in peripheral human CD4^+^CD8^−^ cells.


Mediators of Inflammation 1, 33–37 (1992)

### Trans-active factors controlling the IL-2 gene in adult human T-cell subsets

A. Mouzaki,[1,CA] R.H. Zubler,[1] A. Doucet[1] and D. Rungger[2]

[1]Haematology, Geneva University Hospital, 25, rue Micheli-du-Crest, CH-1211 Geneva 4; [2]Station de Zoologie expérimentale, University of Geneva, 154, rte de Malagnou, CH-1224 Chêne-Bougeries, Switzerland

[CA] Corresponding Author

**IL-2 secretion in total or subsets of PHA/PMA-stimulated PBMC-derived human T-lymphocytes was monitored and found to be largely due to CD4$^+$CD8$^-$ cells. The presence and functional state of transcription factors (TF) was assessed by protein-DNA interaction assays and functional transactivation experiments in the *Xenopus* oocyte system, modulating IL-2 transcription by injection of proteins. The results reveal that CD4$^+$CD8$^-$ cells contain both, functional silencer in their resting, and positive TF in their activated states while the CD8$^+$CD4$^-$ group contains only non-functional positive TF. This demonstrates that the on/off switch of IL-2 transcription is based on the same mechanism in primary T-lymphocytes of mouse spleen and in peripheral human CD4$^+$CD8$^-$ cells.**

**Key words:** Derepression, Functional assay, Silencing, Transactivation, *Xenopus laevis* oocyte

### Introduction

The IL-2 gene is uniquely expressed in stimulated T-cells and its translation product plays a major role in T-cell proliferation. The induction of the IL-2 gene is mediated by a transcriptional enhancer spanning about 300 bp upstream of the cap site (review in Ref. 1). A key promoter element for the tissue-specific transcriptional activation of the IL-2 gene is a purine-rich box (Pud) extending, in the human promoter, from −290 to −263 nucleotides.[1–3] This region is the NF-AT-binding site.[3] If incubated *in vitro* with protein extracts from mouse[2] or human[3] cell lines, it binds a protein which seems to be a positive transcription factor (TF) as it is present in activated cells only.

Using protein extracts from primary mouse splenic T-cells, we were able to show that two different proteins bind to this region at different activity phases of the protein-donor cells. Using functional assays in the *Xenopus laevis* oocyte system, we demonstrated that in resting T-cells the protein which binds to the Pud box is a silencer that represses the basal activity of the IL-2 gene. In mitogen-induced cells a positive TF binds to the same promoter element and efficiently derepresses IL-2 transcription.[4]

In the present study we compared IL-2 secretion by subsets of resting and mitogen-induced human peripheral blood T-cells with the presence and functional state of TFs regulating IL-2 expression. Besides illustrating the resemblance of the regulatory mechanism in mouse and human cells, the results indicate that only CD4$^+$CD8$^-$ cells possess a fully functional IL-2 reglatory mechanism.

### Materials and Methods

*Cells, cultures and control assays:* Heparinized venous blood was collected from adult donors (Transfusion Centre, Div. of Haematology, Geneva University Hospital). MNC were prepared by centrifugation over a Ficoll-Paque gradient (Pharmacia). T-cell- and B-cell-enriched populations were obtained by rosetting with sheep erythrocytes as described.[5] B-lymphocytes were then isolated as described.[5] PB monocytes were obtained by a 2 h plastic adherence step of the remaining MNC following the removal of the majority of the T- and B-lymphocytes. Highly enriched T-cell subsets were isolated from rosette-forming cells using mAb-coated immunomagnetic beads (Dynabeads, Dynal) as recommended by the manufacturers; for the general method see Ref. 6. Briefly, we chose to positively select CD4$^+$CD8$^-$ cells with anti-CD4 mAb and, from the remaining T-cells, CD8$^+$CD4$^-$ cells with anti-CD8 mAb-coated magnetic beads (in each separation using at least five beads/cell). The purity of the positively-selected as well as the remaining T-cell populations was monitored by FACS in an EPICS V Profile Analyser (Coulter Electronics, operated by D. Wohlwend, Geneva Medical Centre) using the following mAb: PE-conjugated mouse anti-human CD4 mAb (DAKO A/S), FITC-conjugated mouse anti-human CD8 mAb (DAKO

© 1992 Rapid Communications of Oxford Ltd.

A/S), FITC-conjugated mouse anti-Leu 12 (CD19) mAb (Becton Dickinson) and FITC-conjugated goat anti-human lg (H and L chains) Ab (Boehringer). If the purity was not satisfactory, the positive selections were repeated on the already selected cells. At least $10^7$ cells are used per group for extraction of putative TFs.

All cells were cultured as described.[5] All T-cell subsets were stimulated by 4h culture in the presence of 5 $\mu$g ml$^{-1}$ PHA and 10 ng ml$^{-1}$ PMA at a density of $10^6$ cells/ml. All cells used in this study were assayed for IL-2 secretion using a bioassay measuring proliferation of CTLL-2 cells as described in Ref 7.

*Salt-extracted S100 proteins (SES100):* The protein extracts used for injections into oocytes were prepared as described,[8] with the modifications introduced in Ref. 4. Proteins attached to chromatin were eluted by adjusting salt concentration to 300 mM before centrifugation at 100 000 × *g*. The protein concentration was determined using the Bradford assay (BioRad).

*Gene clones:* As target gene for the trans-activation experiments we used a construct under the control of four copies of the IL-2 Pud element linked to a truncated thymidine kinase promoter and a chloramphenicol acetyl transferase (CAT) coding unit.[2,4] The activity of this marker gene was measured by CAT assays as described in Ref 9.

*Xenopus oocyte injection:* Oocyte manipulations and injection techniques, including relevant references, are described in detail elsewhere.[10] DNA concentration was chosen on the ground of preliminary experiments which showed that the IL-2 gene displays a basal activity within a range of 1–10 ng of injected DNA per nucleus, with a maximum around 5 ng. To observe both silencing and simulation of transcription, we used 2 ng of IL-2 DNA per oocyte nucleus. The injection protocols employed in this work are: (1) Cytoplasmic injection of proteins, lag of 3 h to allow for migration of nuclear proteins to the germinal vesicle, followed by nuclear injection of DNA. (2) Cytoplasmic injection of proteins, lag of 3 h to allow for migration of nuclear proteins to the germinal vesicle, injection of DNA into the nucleus, followed immediately by a second cytoplasmic injection of the same or different protein extracts. The injected oocytes were incubated for 20–24 h at 20°C and protein was isolated from batches of 10–15 healthy oocytes as described.[8]

*DNA-protein binding assays:* Bandshift analyses were performed as described in Ref 11. Three $\mu$g of protein extracts were incubated with 5 000 cpm (usually about 60–140 pg) DNA radiolabelled probe in the presence of 0.5 $\mu$g poly(dIdC) nonspecific competitor DNA per $\mu$g of protein used. As a probe we used chemically synthesized double stranded oligonucleotides corresponding to the promoter element located between positions −292 and −264 upstream of the IL-2 cap site. Labelling was done by elongation with Klenow polymerase. For competition experiments we used unlabelled Pud oligonucleotide. The competitor was mixed in 20-fold excess with the labelled probe before the addition of the proteins. After 15 min incubation in the presence of the proteins at R.T., the samples were fractionated on 4% nondenaturing polyacrylamide gels at 10 V/cm at R.T.

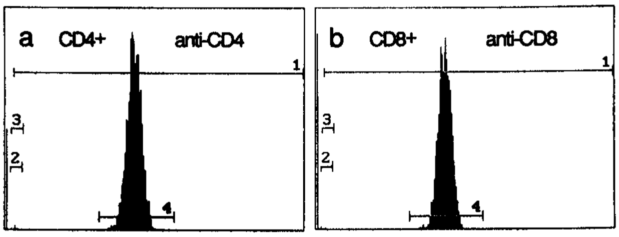

FIG. 1. Purity of T-cell subsets. T-cells were isolated from peripheral blood and subpopulations thereof were purified by positive selection using immunomagnetic beads coated with anti-CD4 or anti-CD8 mAb. FACS analysis confirms purity of 96% for $CD4^+CD8^-$ (a) and 99% for $CD8^+CD4^-$ (b). These results are from a representative experiment.

### Results

*T-cell subsets isolated from PBMC and their ability to secrete IL-2:* Using immunomagnetic beads coated with the appropriate mAb, we were able to isolate by positive selection two subsets of T-cells: $CD4^+CD8^-$ and $CD8^+CD4^-$ (Fig. 1). The average proportion of each T-cell subset in total peripheral T-cells in blood has been determined with samples from 20 donors. It is 50–60% for the $CD4^+CD8^-$ group, 30–40% for $CD8^+CD4^-$ group and 10–20% for the remaining cells reacting with neither mAb (-/-). To ensure that all cells were resting at the

**Table 1.** IL-2 secretion from mitogen-induced peripheral blood T-cell subsets

| Cell type | % purity | IL-2 secretion (U/ml/$10^6$ cells) |
|---|---|---|
| Total T- | 86 | 190 |
| $CD4^+$ | 96 | 102 |
| $CD8^+$ | 95 | 14 |
| $CD8^-CD4^-$ | 90 | 15 |

$E^+$ T-cells were isolated from blood and then $CD4^+$ and $CD8^+$ cells positively selected using mAb-coated magnetic particles. Total T-, $CD4^+$, $CD8^+$ and the remaining non-reactive ($CD4^-CD8^-$) cells were tested for purity by FACS. $10^6$ cells/ml per group were cultured with PHA/PMA for 36 h and then their culture supernatants were collected and assessed for IL-2 biological activity by a proliferation assay of the mouse cytotoxic T-cell line CTLL 2 as described.[7]

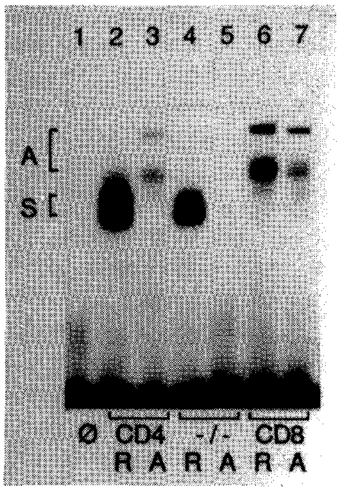

FIG. 2. *In vitro* DNA-protein interaction. Bandshift assays were carried out with a labelled oligonucleotide corresponding to the Pud-element (NF-AT binding site) and with proteins from resting (R) or cells activated (A) for 4h with PHA/PMA. Symbols are: Ø, probe alone; CD4: $CD4^+CD8^-$; CD8: $CD8^+CD4^-$; -/-: $CD4^-CD8^-$ cells. A, left to the panel, indicates the retarded complexes formed by putative positive TFs, S, with silencer proteins.

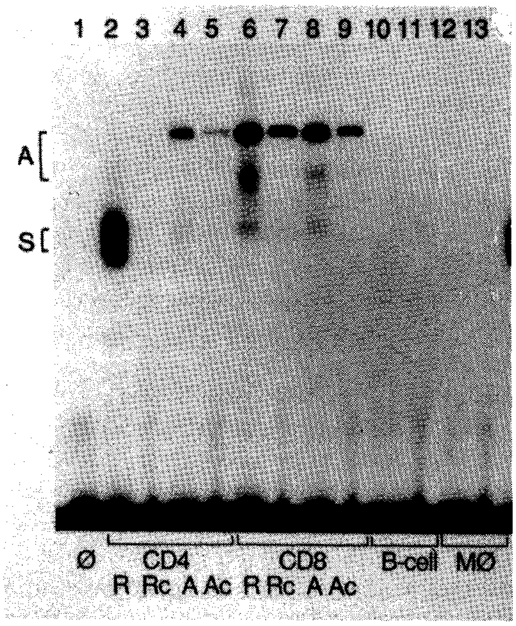

FIG. 3. Specificity of TFs present in T-cell subsets. The same proteins as in Fig. 2 were used. In addition, extracts of B-cells and monocytes (MØ) from two independent donors were tested. Specificity of binding was verified by competition with a 20-fold excess of cold Pud DNA (Ac, Rc). Numbers on top of each plate refer to lanes commented in the text.

beginning of the study, we incubated them O/N in plain culture medium.

Equal amounts of cells from these groups were tested for their ability to secrete IL-2 following stimulation with PHA/PMA, by monitoring the growth induced by addition of their supernatant on CTLL-2 cells. The results (Table 1) show that the $CD4^+CD8^-$ cells are the main secretors while the remaining two subsets secrete only trace amounts of IL-2, provided the low units recorded were not contributed by the few contaminating $CD4^+CD8^-$ cells present in these samples. No IL-2 secretion was detectable in supernatants of B-cells and monocytes (not shown).

*In vitro DNA binding of TFs to the Pud element:* The presence of IL-2 specific TFs in different T-cell subsets and control cells has been assessed by DNA-protein binding (bandshift) assays. Since, in primary mouse T-cells, the main switch of IL-2 regulation, i.e. the switching from negative to positive, acts through the Pud element, we used as a probe for the bandshift experiments an oligonucleotide corresponding to both mouse and human Pud element located at positions −290 to −263 in the latter.[3] Protein extracts from resting or mitogen-stimulated cells recovered from the different cell populations were tested for the presence of proteins that bind *in vitro* to this control element.

The results (Figs 2 and 3) show that resting $CD4^+CD8^-$ cells contain large amounts of protein that form two bands migrating closely together (Fig. 2, lane 2). By comparison with the relative migration of DNA-protein complexes from mouse material,[4] these bands may be formed by silencer-like proteins. Indeed, upon mitogenic stimulation they disappear, and other Pud-binding proteins appear that form two slower migrating complexes (lane 3) that are most probably due to positive TFs. The resting double negative cells contain silencer-like molecules (lane 4) but form no positive TF upon activation (lane 5). By contrast, both resting and mitogen stimulated $CD8^+CD4^-$ cells form only activator-like complexes (lanes 6 and 7).

As far as the above proteins are concerned, Fig. 3 gives similar results and, in addition, shows that binding of the silencer to the labelled Pud element is fully competed out by the addition of a 20-fold excess of cold Pud oligonucleotides (cf. lanes 2/3). Also the lower of the positive TF complexes is efficiently competed, whereas the slow migrating complex may be nonspecific as it is poorly competed out (cf. lanes 4/5, 6/7, 8/9). Figure 3 also shows that B-cells (lanes 10/11) and monocytes (lanes 12/13) completely lack Pud-specific DNA binding proteins.

*Functional tests of TFs in the Xenopus oocyte: In vitro* DNA-binding of a protein, as monitored in bandshift experiments, does not necessarily imply that the binding factor is functionally active *in vivo.* Therefore, the same protein extracts isolated from the different cell types were also assayed for their functional activity in the *Xenopus* oocyte. Using this system, we have previously shown[4] that proteins from resting mouse T-cells efficiently silence basal transcription of the injected IL-2 gene, whereas proteins from mitogen-activated cells do not

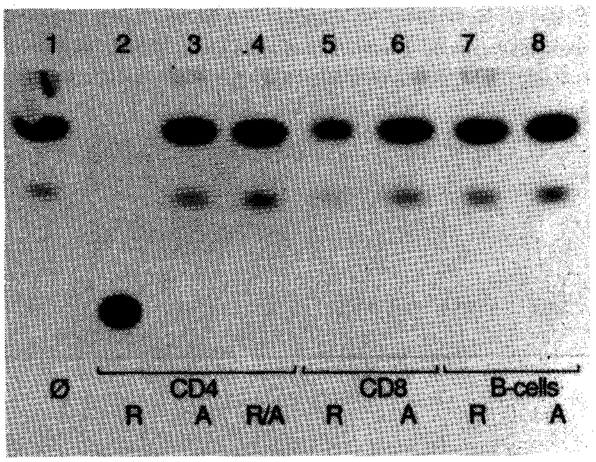

FIG. 4. Functional tests of TFs in the oocyte system. Proteins (5 ng) from the different T-cell subsets in their resting (R) or activated (A) phase, were injected into the oocyte cytoplasm, followed by injection of 2 ng of 4 × Pud-CAT gene constructs into the oocyte nucleus. For the $CD4^{+}CD8^{-}$ group, following injection of silencer and the gene, extract from activated cells was added in a third, cytoplasmic, injection to test for derepression (R/A). The symbols used for protein extracts are the same as in Fig. 2. CAT assays yield three forms of acetylated chloramphenicol (lowest spot, not converted; second spot, 1-; third, 3-, fourth; 1-3-acetylation). Activity was measured on aliquots corresponding to one oocyte taken from lysates produced from 10–15 oocytes.

silence, but fully derepress previously silenced genes.

The tests (Fig. 4) which were carried out with a target gene composed of 4X the Pud promoter element and a CAT coding segment, show that the silencer in resting $CD4^{+}CD8^{-}$ cells is fully effective in suppressing the basal activity of this promoter (Fig. 4, cf. lanes 1 and 2). Activated $CD4^{+}CD8^{-}$ cells contribute a positive TF that does not silence basal activity (lane 3) and efficiently derepresses the IL-2 promoter silenced by a first dose of resting cell extracts (lane 4). As expected, resting $CD8^{+}CD4^{-}$ cells that do not contain silencer-like binding factors, are not repressing basal IL-2 transcription. Though some of the sample is lost, full chloramphenicol conversion can still be observed (lane 5). Not surprisingly, no silencing activity is exerted by B-cell extracts (lanes 7 and 8).

### Discussion

IL-2 expression was monitored in distinct subsets of T-cells in human peripheral blood and compared with the presence and functional state of TFs controlling the off/on switch in IL-2 gene transcription. Our data support the mainstream notion that the major IL-2 secretors are the activated $CD4^{+}CD8^{-}$ cells and show that they are the only cells to possess both functional silencer and positive TF controlling the IL-2 gene.

In a previous study, we showed that in mouse resting splenic T-cells the IL-2 gene is silenced through the attachment of a silencer protein on the purine-rich box situated between positions −292 to −264 upstream of the cap site and that, upon cellular induction, a positive TF derepresses the gene.[4] We looked for these regulatory factors in human T-cells from peripheral blood and found that they exist and also bind to the Pud promoter element. Comparing the formation of DNA-protein complexes by extracts from resting and mitogen-stimulated cells, and their functional activity in the transcription assay with our former observations using mouse material, we may conclude that the faster migrating complex(es) in the band shift assays are formed by silencer molecules, whereas the two slower migrating complexes comprise positive TFs. Whether we observe here the two subunits of the NF-AT described recently by Flanagan *et al.*,[12] remains to be seen. What can be ascertained, is that none of these positive TFs (or the silencer) appear in B-cells or monocytes and they thus seem to be tissue-specific.

Interestingly, only the $CD4^{+}CD8^{-}$ contain both a functional silencer in their resting state and an active positive TF following activation. The $CD8^{+}CD4^{-}$ cells that contain only positive TF-like molecules in either activity phase, are not secreting IL-2 and this TF seems thus not to be functional. In view of recent findings on positive TF function involving controlled nuclear targeting,[12] it is not easy to verify, whether the positive TF-like protein present in these cells is functional alone, whether it is in an inactive configuration or whether a normal silencer is needed to help building up positive pre-initiation complexes. Such questions will be addressed in a later study.

Conclusively, we have shown that, among the peripheral T-cells in human adult blood, only the $CD4^{+}CD8^{-}$ helper inducer cells contain both functional components regulating IL-2 transcription. This mechanism, as shown here for the first time for human IL-2, includes a silencer present in resting cells that inactivates the gene by binding to the Pud element, and positive TFs derepressng the IL-2 promoter by interacting to the same element.

### References

1. Crabtree GR. Contigent genetic regulatory events in T-lymphocyte activation. *Science* 1989; **243:** 355–361.
2. Serfling E, Barthelmäs R, Pfeuffer I, *et al.* Ubiquitous and lymphocyte-specific factors are involved in the induction of the mouse interleukin-2 gene in T-lymphocytes. *EMBO J* 1989; **8(2):** 465–473.
3. Shaw J-P, Utz PJ, Durand DB, *et al.* Identification of a putative regulator of early T-cell activation genes. *Science* 1988; **241:** 202–205.
4. Mouzaki A, Weil R, Muster L, *et al.* Silencing and trans-activation of the mouse IL-2 gene in *Xenopus* oocytes by proteins from resting and mitogen-induced primary T-lymphocytes. *EMBO J* 1991; **10(6):** 1399–1406.
5. Tucci A, Mouzaki A, James H, *et al.* Are cord blood B-cells functionally mature? *Clin Exp Immunol* 1991; **84:** 389–394.
6. Gaudernack GG, Leivestad T, Ugelstad J, *et al.* Isolation of pure functionally active $CD8^{+}$ T-cells. Positive selection with monoclonal antibodies directly conjugated to monosized magnetic microspheres. *J Immunol Methods* 1986; **90:** 179–187.
7. Gillis S, Smith KA. Long term culture of tumor-specific cytotoxic T-cells. *Nature* 1977; **268:** 154–156.
8. Rungger D, Muster I, Boeck R, *et al.* Tissue-specific trans-activation of the rabbit-b-globin promoter in *Xenopus* oocytes. *Differentiation* 1990; **44:** 8–17.

9. Gorman CM, Moffat LF, Howard BH. Recombinant genomes which express chloramphenicol acetyltransferase in mammalian cells. *Mol Cell Biol* 1982; **2(9)**: 1044–1051.
10. Bertrand D, Valera S, Cooper E, *et al.* Electrophysiology of neuronal nicotinic acetylcholine receptors expressed in *Xenopus* oocytes following nuclear injection of genes or cDNAs. In: Conn PM, ed. *Methods in Neuroscience, (Electrophysiology and microinjection), Volume 4.* Academic Press Inc; San Diego, 1991: 174–193.
11. Schreiber E, Matthias P, Müller MM, *et al.* Identification of a novel specific octamer binding protein (OTF-2B) by proteolytic clipping bandshift assay (PCBA). *EMBO J* 1988; **7(13)**: 4221–4229.
12. Flanagan WM, Corthésy B, Bram RJ, *et al.* Nuclear association of a T-cell transcription factor blocked by FK-506 and cyclosporin A. *Nature* 1991; **352:** 803–806.

ACKNOWLEDGEMENTS. This work was funded by the Swiss National Foundation grants 3.128654.90 (DR) and 31.26502.89 (RHZ).

**Received 16 October 1991; accepted 22 October 1991**

## References

[PDF_00379] Crabtree G. R. (1989). Contingent genetic regulatory events in T lymphocyte activation.. Science.

[PDF_00412] Flanagan W. M., Corthésy B., Bram R. J., Crabtree G. R. (1991). Nuclear association of a T-cell transcription factor blocked by FK-506 and cyclosporin A.. Nature.

[PDF_00391] Gaudernack G., Leivestad T., Ugelstad J., Thorsby E. (1986). Isolation of pure functionally active CD8+ T cells. Positive selection with monoclonal antibodies directly conjugated to monosized magnetic microspheres.. J Immunol Methods.

[PDF_00395] Gillis S., Smith K. A. (1977). Long term culture of tumour-specific cytotoxic T cells.. Nature.

[PDF_00401] Gorman C. M., Moffat L. F., Howard B. H. (1982). Recombinant genomes which express chloramphenicol acetyltransferase in mammalian cells.. Mol Cell Biol.

[PDF_00386] Mouzaki A., Weil R., Muster L., Rungger D. (1991). Silencing and trans-activation of the mouse IL-2 gene in Xenopus oocytes by proteins from resting and mitogen-induced primary T-lymphocytes.. EMBO J.

[PDF_00397] Rungger D., Muster L., Boeck R., Nichols A. (1990). Tissue-specific trans-activation of the rabbit beta-globin promoter in Xenopus oocytes.. Differentiation.

[PDF_00409] Schreiber E., Matthias P., Müller M. M., Schaffner W. (1988). Identification of a novel lymphoid specific octamer binding protein (OTF-2B) by proteolytic clipping bandshift assay (PCBA).. EMBO J.

[PDF_00381] Serfling E., Barthelmäs R., Pfeuffer I., Schenk B., Zarius S., Swoboda R., Mercurio F., Karin M. (1989). Ubiquitous and lymphocyte-specific factors are involved in the induction of the mouse interleukin 2 gene in T lymphocytes.. EMBO J.

[PDF_00384] Shaw J. P., Utz P. J., Durand D. B., Toole J. J., Emmel E. A., Crabtree G. R. (1988). Identification of a putative regulator of early T cell activation genes.. Science.

[PDF_00389] Tucci A., Mouzaki A., James H., Bonnefoy J. Y., Zubler R. H. (1991). Are cord blood B cells functionally mature?. Clin Exp Immunol.

